# Clearance of Apoptotic Cells by Macrophages Induces Regulatory Phenotype and Involves Stimulation of CD36 and Platelet-Activating Factor Receptor

**DOI:** 10.1155/2013/950273

**Published:** 2013-11-20

**Authors:** Matheus Ferracini, Francisco J. O. Rios, Mateus Pecenin, Sonia Jancar

**Affiliations:** ^1^Department of Immunology, Biomedical Sciences Institute, University of São Paulo, Avenue Professor Lineu Prestes 1730, ICB IV, Sala 140/146, 05508-000 São Paulo, SP, Brazil; ^2^Institute of Cardiovascular and Medical Sciences, British Heart Foundation Glasgow Cardiovascular Research Centre, University of Glasgow, Glasgow G12 8TA, UK

## Abstract

Phagocytosis of apoptotic cells (efferocytosis) induces macrophage differentiation towards a regulatory phenotype (IL-10^high^/IL-12p40^low^). CD36 is involved in the recognition of apoptotic cells (AC), and we have shown that the platelet-activating factor receptor (PAFR) is also involved. Here, we investigated the contribution of PAFR and CD36 to efferocytosis and to the establishment of a regulatory macrophage phenotype. Mice bone marrow-derived macrophages were cocultured with apoptotic thymocytes, and the phagocytic index was determined. Blockage of PAFR with antagonists or CD36 with specific antibodies inhibited the phagocytosis of AC (~70–80%). Using immunoprecipitation and confocal microscopy, we showed that efferocytosis increased the CD36 and PAFR colocalisation in the macrophage plasma membrane; PAFR and CD36 coimmunoprecipitated with flotillin-1, a constitutive lipid raft protein, and disruption of these membrane microdomains by methyl-**β**-cyclodextrin reduced AC phagocytosis. Efferocytosis induced a pattern of cytokine production, IL-10^high^/IL-12p40^low^, that is, characteristic of a regulatory phenotype. LPS potentiated the efferocytosis-induced production of IL-10, and this was prevented by blocking PAFR or CD36. It can be concluded that phagocytosis of apoptotic cells engages CD36 and PAFR, possibly in lipid rafts, and this is required for optimal efferocytosis and the establishment of the macrophage regulatory phenotype.

## 1. Introduction

Clearance of apoptotic cells (AC) by macrophages, also called efferocytosis, plays a central role in tissue homeostasis, and the impaired clearance of altered cells has been associated with the development of autoimmune and chronic inflammatory diseases [1]. Macrophages can acquire distinct phenotypes depending on the stimulus. M1 or classically activated macrophages are induced by the recognition of PAMPs (pathogen associated molecular patterns) and by proinflammatory cytokines. These cells exhibit high microbicidal activity and induce inflammation. In contrast, M2 macrophages produce anti-inflammatory and tissue remodelling cytokines and can be divided into subtypes according to the stimulus; the alternatively activated macrophages are induced by Th2 cytokines, and the regulatory macrophages are induced by anti-inflammatory cytokines, immune complexes, apoptotic cells, and oxidised lipids, among other stimuli [2, 3].

The first evidence that efferocytosis induces a suppressor phenotype came from the studies of Fadok et al. [4]. The authors showed that the addition of apoptotic cells to macrophages inhibited the expression of proinflammatory cytokines induced by LPS. Later on, it was reported that the clearance of apoptotic neutrophils induced an IL-10^high^/IL-12^low^ regulatory phenotype in macrophages [5].

There is evidence that platelet-activating factor (PAF) or PAF-like moieties present in the tumour microenvironment promote tumour growth by suppressing macrophage functions [6]. The addition of apoptotic cells to a subtumorigenic dose of melanoma cells promoted tumour growth, and this phenomenon was significantly reduced when an antagonist of the PAF receptor (PAFR) was injected into the tumour site [7]. Also, phagocytosis of AC has been shown to be decreased by the pretreatment of macrophages with antagonists of PAFR [8]. Moreover, PAFR was shown to be involved in the systemic immunosuppression induced by environmental stressor agents, such as UV radiation and cigarette smoke [9, 10]. 

The membrane of AC exhibits oxidised phospholipids, which are not present in viable cells. Macrophages recognise these modified lipids through a group of receptors known as “scavenger receptors” [11]. It is well established that CD36 is one of the main “scavenger” receptors involved in the recognition of modified lipids, including oxidised LDL (oxLDL) [11, 12]. We showed cross-desensitisation between oxLDL and PAF [8] and that engagement of both CD36 and PAFR is required for the optimal uptake of oxLDL [13, 14]. In the present study, we investigated the contribution of each receptor for phagocytosis of AC and for the induction of a regulatory phenotype (IL-10^high^/IL-12p40^low^) in murine bone marrow-derived macrophages.

## 2. Materials and Methods

### 2.1. Cell Culture

Male, 6–8-week-old C57BL/6 mice were obtained from our own animal facilities and were housed in a 12 h light/dark cycle room with water and food *ad libitum*. Animal care and research protocols were in accordance with the principles and guidelines adopted by the Brazilian College of Animal Experimentation (COBEA) and approved by the Biomedical Sciences Institute/USP-Ethical Committee for Animal Research (CEEA). Bone marrow-derived macrophages (BMDM) were obtained as previously described by Davies and Gordon [15], with minor modifications. In brief, femurs were flushed with DMEM (Dulbecco's modified eagle medium containing 2 mM l-glutamine, 100 U/mL penicillin G, and 100 mg/mL streptomycin, all from Gibco, Long Island, NY, USA), using a 26-G x 1/2′′ needle. Cells were grown in DMEM containing 20% LCM (L-929 cell conditioned medium) and 15% of heat-inactivated foetal calf serum (FCS), incubated at 37°C in 5% CO_2_. On day 3, new fresh DMEM with LCM was added. A monolayer of macrophages was scraped on day 6 (96% of the cells were positive for CD11b and F4/80). Macrophages were cultured in DMEM with 5% FCS for one day before the experiments.

### 2.2. Induction of Apoptosis

Apoptosis of thymocytes was performed as described previously [16]. Briefly, thymuses from young (5-6 weeks old) male C57BL/6 mice were harvested and macerated. A suspension of thymocytes at a concentration of 1 × 10^6^ cells/mL was incubated with 1 *μ*M of dexamethasone (Sigma-Aldrich, St. Louis, MO, USA) for 6 h in DMEM with 10% FCS. Before addition to the macrophages, apoptotic thymocytes were washed three times for the removal of dexamethasone.

### 2.3. Cell Treatment

The blockage of PAFR was performed using the chemically unrelated antagonists WEB (WEB2086 from Tocris, Bristol, UK) and CV (CV3988 from Enzo Lifesciences, Farmingdale, NY, USA), which were added to macrophages 30 min prior to the addition of apoptotic cells. The concentration of WEB (50 *μ*M) and CV (10 *μ*M) used here was based on previous works by our group [8, 17]. CD36 was blocked by a specific anti-CD36 blocking antibody (monoclonal IgA anti-CD36, clone CRF D-2712, BD Biosciences, Franklin Lakes, NJ, USA) at 1 *μ*g/mL, added 30 min before the addition of AC. The disruption of lipid rafts was performed by depleting cholesterol from membranes with 1 mM methyl-*β*-cyclodextrin (*β*CD) or with the inactive analogue *α*-cyclodextrin (*α*CD) (all from Sigma-Aldrich, St. Louis, MO, USA) 10 min prior to AC.

### 2.4. Phagocytosis of Apoptotic Cells

To determine the phagocytic index, macrophages were allowed to adhere to glass coverslips inside 24-well culture microplates. On the next day, nonadherent cells were removed, macrophages were treated with drugs or blocking antibody (WEB, CV, *β*CD, *α*CD, vehicle or CD36 Ab, and control Ab), and 10 AC per macrophage were added for phagocytosis for 90 min. The noningested cells were removed and coverslips were stained with haematoxylin and eosin. Results are expressed as a phagocytic index, which was derived by multiplying the percentage of macrophages containing at least one ingested AC by the number of targets ingested by the macrophages.

### 2.5. Coimmunoprecipitation and Immunoblotting

Macrophages were treated with PAF (10^−7^ M) (Cayman Chemical, Ann Arbor, Michigan, USA) or apoptotic cells (10 per macrophage) for 20 min. After washing and resting, activated cells were lysed without agitation on ice for 30 min using HEPES buffer containing 1 mM CaCl_2_, 1 mM MgCl_2_, 1% Triton X-100, protease inhibitor cocktail (Sigma-Aldrich, St. Louis, MO, USA), and phosphatase inhibitors (NaF and Na_3_VO_4_ (Calbiochem-Merck Chemicals, Nottingham, UK). Lysates were incubated overnight with the primary antibody of interest (rabbit anti-PAFR or mouse IgA anti-CD36) at 4°C with gentle agitation. Protein A-Sepharose (GE Healthcare, NJ, USA) and protein G-Sepharose (Amersham-Pharmacia Biotech, Uppsala, Sweden) were added to samples containing anti-PAFR or anti-CD36, respectively, and incubated for 3 h at 4°C with gentle agitation. Immune complexes bound to beads were washed three times with HEPES buffer containing protease and phosphatase inhibitors, without Triton X-100, and boiled in SDS sample buffer for 5 minutes. Proteins were separated by 10% SDS-PAGE, transferred to a Hybond nitrocellulose membrane (GE Healthcare, NJ, USA), and incubated with rabbit-anti-PAFR (Cayman Chemical, Ann Arbor, Michigan, USA), mouse IgA-anti-CD36 (BD Biosciences, Franklin Lakes, NJ, USA) or mouse anti-flotillin-1 (BD Biosciences, Franklin Lakes, NJ, USA). As secondary antibodies, we used anti-rabbit IgG-HPR (1 : 2000), anti-mouse-HRP (1 : 1000) (Cell Signaling Technology, Beverly, MA, USA), biotin-anti-IgA (1 : 500) (BD Biosciences, Franklin Lakes, NJ, USA) with streptavidin-HRP (1 : 200) (Life Technologies, Carlsbad, CA, USA) and visualised using SuperSignal West Pico Chemiluminescent Substrate (Thermo Scientific, Rockford, IL, USA). The resulting autoradiograms were analysed with the AlphaEaseFC software V3.2 beta (Alpha Innotech, San Leandro, CA, USA).

### 2.6. Confocal Microscopy

Macrophages were plated on glass cover slips and treated with apoptotic cells (10 per macrophage) for 20 minutes. After gentle washing with PBS, cells were fixed with 3% paraformaldehyde. Cells were blocked with 1% BSA in PBS before incubation with primary antibodies including anti-PAFR (1 : 100) (Cayman Chemical, Ann Arbor, Michigan, USA) and IgA anti-CD36 (1 : 100) (BD Biosciences, Franklin Lakes, NJ, USA). Alexa Fluor 647 goat anti-rabbit IgG (1 : 100) (Invitrogen-Life Technologies, Carlsbad, CA, USA) and biotin-anti-mouse IgA (1 : 200) with streptavidin-PE (1 : 200) (BD Biosciences, Franklin Lakes, NJ, USA) were used as secondary antibodies. Cells stained with secondary antibody and control antibody were used to control for the background from each fluorophore. Slides were mounted in Prolong Gold antifade reagent with DAPI (4,6-diamidino-2-phenylindole) (Invitrogen-Life Technologies, Carlsbad, CA, USA). Cells were imaged on a Zeiss LSM 510 confocal microscope using the 100x oil objective. Confocal images were taken with identical settings to allow the comparison of staining. Single confocal sections of the cells were captured in multitrack. Each set of frames from a given treatment condition depicts a representative from at least 20 analysed cells in three independent experiments. Colocalisation was quantified by analysing at least 10 images and determining Pearson's coefficient using JACoP (Just Another Colocalisation Plugin), and the software ImageJ 1.46r (NIH, USA), which is available at http://imagej.nih.gov/ij/ [18]. Basically, a linear equation is calculated to describe the relationship between the intensities in two images (separate colours). The slope of this linear approximation provides the rate of association of two fluorochromes, and Pearson's coefficient estimates the goodness of this approximation [19].

### 2.7. Cytokine Measurement

Macrophages were treated with drugs/blocking antibody (WEB, CV, *β*CD, *α*CD, or CD36 Ab, 30 min before addition of AC (10 per macrophage)). After 24 h of phagocytosis, one group was stimulated with LPS (10 ng/mL) for 24 h. The supernatants were centrifuged for the removal of noningested cells, and levels of IL-10 and IL-12p40 were measured using a BD OptEIA kit (BD Biosciences, San Diego, CA, USA) according to the manufacturer's specifications.

### 2.8. Statistical Analysis

Data are presented as mean ± standard error mean (SEM). Analysis of variance (ANOVA) and the Student-Newman-Keuls posttest were used to evaluate the statistical significance of the differences between three or more groups. A two-tailed unpaired Student's *t*-test was used when differences between two groups were analysed. Significance was assumed when *P* < 0.05.

## 3. Results

### 3.1. Efferocytosis Is Dependent on the Engagement of PAFR and CD36 

**Figure 1:**
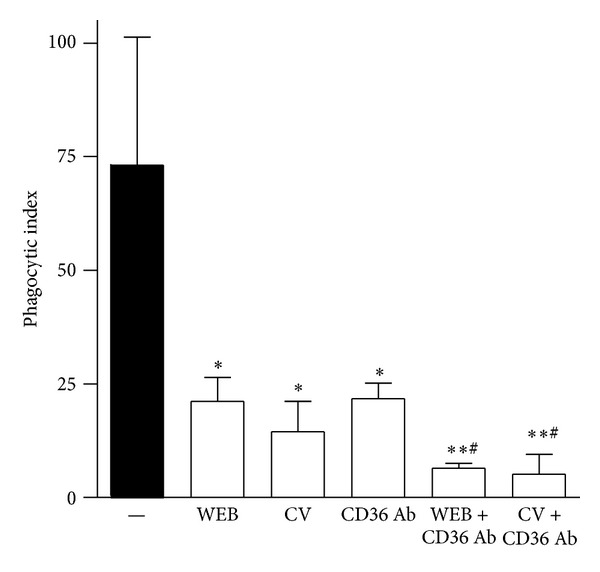
Efferocytosis involves PAFR and CD36. BMDM plated on coverslips were treated with PAFR antagonists WEB (WEB2086, 50 *μ*M) or CV (CV3988, 10 *μ*M) alone or in association with a specific blocking antibody to CD36 (1 *μ*g/mL) for 30 min before addition of apoptotic thymocytes (10 per macrophage). After 90 min of phagocytosis, cells were washed for removal of noningested targets and coverslips were stained with haematoxylin/eosin. The phagocytic indexes (derived by multiplying the percentage of macrophages containing at least one ingested target by the number of targets ingested by the macrophages) were assessed by cell counting under an optical microscope. Values are mean ± SEM of at least three independent experiments (*P < 0.05 versus control; **P < 0.01 versus control; ^#^ *P* < 0.05 versus WEB, CV, and CD36 Ab).

To investigate whether the engagement of both PAFR and CD36 is required for macrophages to phagocytose AC, murine macrophages were pretreated with two chemically unrelated antagonists, WEB2086 and CV3988, and CD36 was blocked by a specific antibody.  Figure 1 shows that PAFR antagonists decreased the phagocytosis of AC (WEB 71% and CV 79%). Blockage of CD36 also reduced the phagocytosis of AC (70%). The simultaneous blockage of CD36 and PAFR was even more effective at inhibiting the phagocytosis of AC (90 and 93% for association of CD36 with WEB2086 and CV3988, resp.). These results suggest that both receptors are involved in the phagocytosis of AC by macrophages.

**Figure 2:**
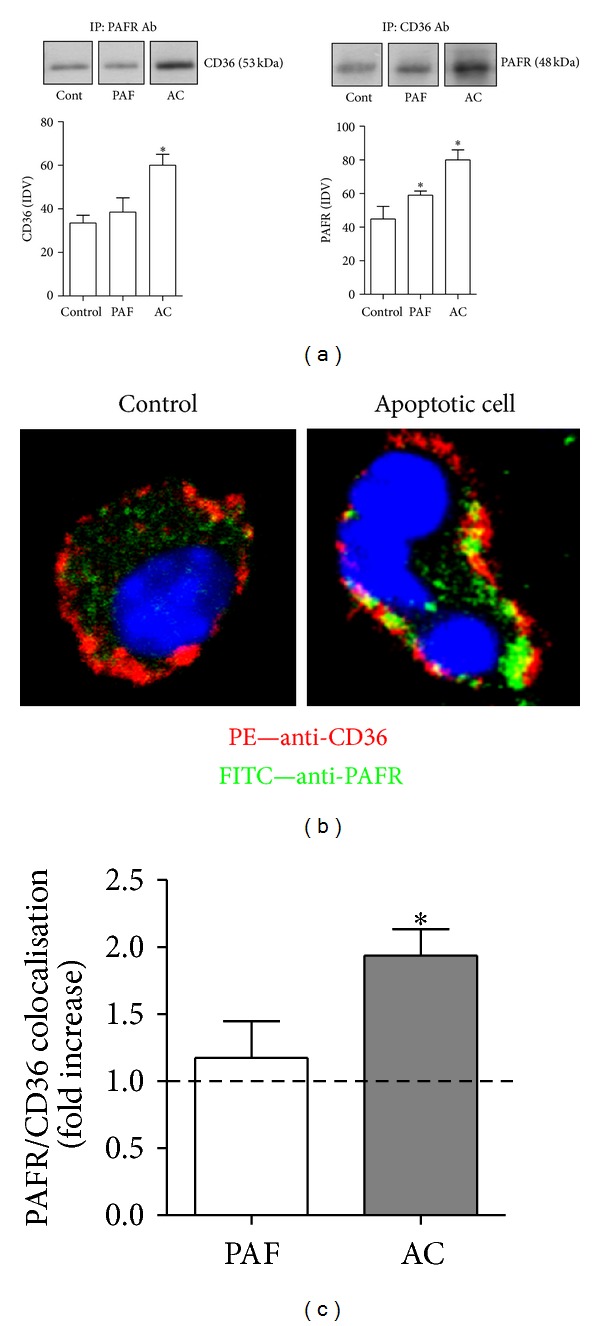
Colocalisation of PAFR and CD36 occurs during efferocytosis. BMDM were treated with PAFR agonist (PAF, 10^−7^ M) or apoptotic thymocytes (10 per macrophage) for 20 min to assess coimmunoprecipitation and colocalisation of PAFR and CD36. After washing, cells were lysed and subjected to immunoprecipitation and immunoblotting as described in Section 2, using antibodies to CD36 and PAFR (a). Another group was subjected to fixation prior to staining with anti-PAFR and anti-CD36 primary antibodies, followed by FITC- and PE-labelled secondary antibodies, respectively, and visualised by confocal microscopy as described in Section 2 (b). Quantification of colocalisation (c) was performed using Pearson's coefficient and JACoP/ImageJ software, and data are presented as mean ± SEM of 15 pictures from three independent experiments (*P < 0.05 versus vehicle). Protein expression was quantified by the AlphaEaseFC software v3.2 beta (Alpha Innotech). The autoradiographs show one representative experiment, and graph data are presented as mean ± SEM of three experiments (*P < 0.05 versus control).

We then investigated the possibility of physical interaction between these receptors by evaluating whether phagocytosis of apoptotic cells induces the coimmunoprecipitation and colocalisation of CD36 with PAFR. We found that the addition of AC to macrophages induced the immunoprecipitation of PAFR and CD36, detected within 20 min (Figure 2(a)). A basal coprecipitation of PAFR and CD36 was also observed in resting (control) macrophages, which was not increased after the stimulation of PAFR by the agonist PAF alone. This was reinforced by colocalisation analysis performed by confocal microscopy. Macrophages incubated with AC were labelled with antibodies to CD36 (red) and PAFR (green). We found in AC-treated macrophages, in contrast to the control and PAF-treated macrophages, that there was a redistribution of PAFR and CD36, increasing their colocalisation (2-fold), as shown by the enhanced yellow spots on the macrophage plasma membrane, visualised in Figure 2(b) and quantified in Figure 2(c). As the samples were not permeabilised, the CD36 and PAFR fluorescence observed reflects the presence of these receptors in the cell membrane. Based on this, we can conclude that the overlap of CD36 and PAFR observed in Figure 2(b) occurs on the cell membrane. These data strongly suggest that AC induces a spatial redistribution of PAFR and CD36 in the plasma membrane, resulting in increased immunoprecipitation and colocalisation of these receptors.

**Figure 3:**
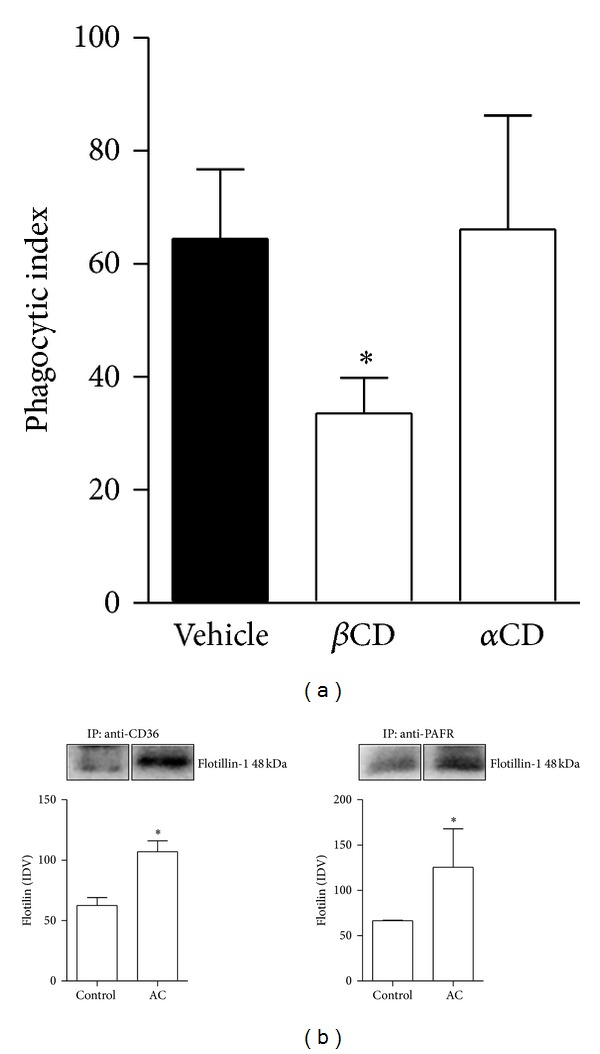
Efferocytosis requires lipid raft integrity. BMDM were treated with *β*CD (1 mM) or *α*CD (1 mM) for 10 min before addition of apoptotic thymocytes (10 per macrophages) for 90 min for phagocytosis. After washing for removal of noningested targets, coverslips were stained with haematoxylin/eosin and the phagocytic indexes (derived by multiplying the percentage of macrophages containing at least one ingested target by the number of targets ingested by the macrophages) were assessed by cell counting under an optical microscope. Values are mean ± SEM of at least three independent experiments (*P < 0.05 versus control) (a). In parallel, BMDM were incubated with apoptotic thymocytes (10 per macrophage) for 20 min before addition of lysis buffer. Cells lysates were subjected to immunoprecipitation/immunoblotting assays as described in Section 2 using antibodies to PAFR or CD36 and flotillin-1. Protein expression was quantified by the AlphaEaseFC software V3.2 beta. The autoradiographs show one representative experiment, and graph data are presented as mean ± SEM of three experiments (*P < 0.05) (b).

Lipid rafts are specialised microdomains in the plasma membrane that allow interactions between receptors. To investigate whether intact lipid rafts are required for the phagocytosis of AC, the phagocytic index was assessed after the treatment of macrophages with methyl-*β*-cyclodextrin (*β*CD), which depletes cholesterol from the membrane, causing a disruption of lipid rafts. We found that the ingestion of apoptotic cells was reduced after macrophages were treated with *β*CD (Figure 3(a)), unlike macrophages that were treated with alpha-cyclodextrin (*α*CD; chemically-related to *β*CD, used as a control with no effect in lipid rafts). Furthermore, immunoprecipitation assays were performed for the detection of flotillin-1, which is a protein that is typically found in lipid rafts [20].  Figure 3(b) shows that, in macrophages that have ingested apoptotic cells, the amount of flotillin-1 was significantly increased in the samples immunoprecipitated with antibodies to CD36. Likewise, in the samples immunoprecipitated with antibodies to PAFR, the amount of flotillin-1 was also increased. Together, these results show that when apoptotic cells interact with macrophages, PAFR and CD36 are recruited to the lipid rafts and that the integrity of these membrane domains is required for the optimal ingestion of apoptotic cells.

### 3.2. PAFR and CD36 Contribute to the Induction of a Regulatory Phenotype in Macrophages during Efferocytosis

**Figure 4:**
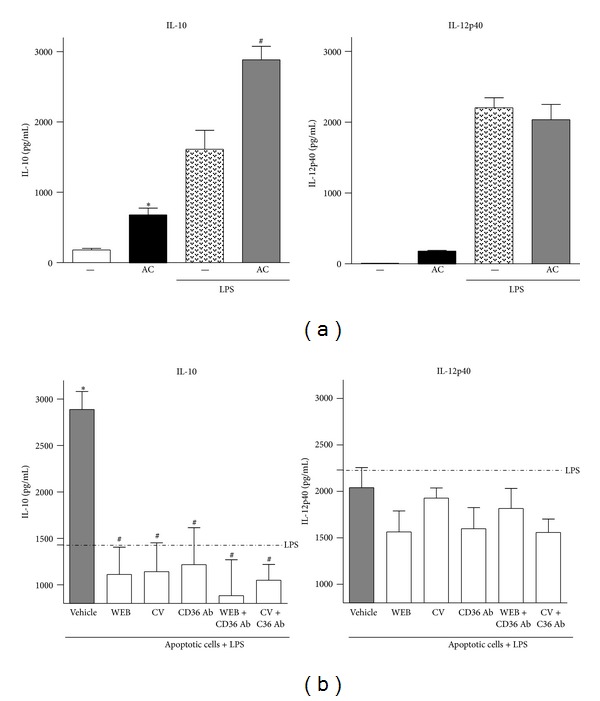
Efferocytosis-induced IL-10 and IL-12p40 production. Apoptotic thymocytes were added to BMDM (10 per macrophage), and after 24 h the supernatants were assayed (a). Another group, after 24 h of contact with apoptotic cells, was stimulated with LPS (10 ng/mL), and supernatants were assayed after 24. Treatments with PAFR antagonists WEB (WEB2086, 50 *μ*M) or CV (CV3988, 10 *μ*M) and with specific blocking antibody to CD36 (1 *μ*g/mL) were performed 30 min prior to the addition of apoptotic thymocytes. IL-10 and IL-12p40 levels in the cultures supernatants were assessed by ELISA according to manufacturer's specifications. Data are presented as the mean ± SEM (*P < 0.05 versus control without AC; ^#^ *P* < 0.05 versus control with apoptotic cells).

We next evaluated the phenotype of macrophages that have ingested AC by measuring the production of IL-12p40 and IL-10.  Figure 4(a) shows that these macrophages produced IL-10 and small amounts of IL-12p40. In order to potentiate the production of these cytokines, LPS was added to macrophages 24 h after efferocytosis. In these conditions, the production of IL-10, increased whereas IL-12p40 was not affected, resulting in an IL-10^high^/IL-12^low^ phenotype, which is characteristic of regulatory macrophages. We then evaluated the effect of PAFR antagonists, anti-CD36 antibodies, or the combination of both on cytokine production.  Figure 4(b) shows that the blockage of PAFR or CD36 inhibited efferocytosis-induced IL-10 production without affecting IL-12p40. In the case of treatment with the PAFR antagonist CV3988, the IL-10/IL-12p40 ratio changed from 1.4 in the nontreated to 0.6 in the antagonist-treated macrophages. Blocking CD36 or both PAFR and CD36 had a similar effect, redirecting macrophages towards a more activated profile by favouring higher amounts of IL-12p40 relative to IL-10. These results show that, during efferocytosis, macrophages acquire an IL-10^high^/IL-12p40^low^ cytokine production profile and that both PAFR and CD36 are involved in the establishment of a regulatory phenotype in macrophages that have ingested AC.

## 4. Discussion

Here, we showed that engagement of both PAFR and CD36 is required for the phagocytosis of apoptotic cells and for the induction of the IL-10^high^/IL-12p40^low^ profile (regulatory) that follows efferocytosis. In addition, we showed that efferocytosis increased the colocalisation of these receptors and immunoprecipitation with the membrane lipid raft marker protein flotillin-1; also we demonstrated that lipid raft integrity is required for the optimal ingestion of AC. To our knowledge, this is the first study to suggest that PAFR and CD36 co-localise and are recruited to lipid raft microdomains during efferocytosis and that the engagement of both receptors is required for AC ingestion and the induction of a regulatory phenotype of macrophages.

Both PAFR and CD36 recognise modified/oxidised lipids and share common ligands, such as oxLDL and moieties present in the membrane of apoptotic cells, as shown here. Recently, we showed that co-stimulation of PAFR and CD36 is required for the uptake of oxLDL and for the induction of cytokine genes by oxLDL [13, 14]. Here, we showed that recognition and phagocytosis of apoptotic cells were also dependent on PAFR and CD36. This was shown by using two chemically unrelated PAFR antagonists and by blocking CD36 with a specific antibody. Interestingly, the simultaneous blockage of both receptors had an additive effect in efferocytosis inhibition. These results suggest that moieties present in the AC membrane engage both receptors, and that this is required for the ingestion of AC. Furthermore, we were able to demonstrate by immunoprecipitation and confocal microscopy that efferocytosis caused an increase in CD36 and PAFR colocalisation. Since the samples were not permeabilised, we can conclude that the overlap of CD36 and PAFR occurs in the cell membrane. However, the possibility of interactions between receptors in the phagosome membrane after apoptotic cell ingestion or even on cytoplasmic compartments cannot be ruled out.

The dynamics and trafficking of membrane receptors involve the formation of lipid rafts (LR), which are cholesterol- and sphingomyelin-rich membrane microdomains that function as platforms to promote the association of signalling molecules and the compartmentalisation of cellular processes [21]. It has been shown that CD36 is recruited to lipid rafts in a ligand-dependent manner [22, 23], and that PAFR has a binding motif for a constitutive protein of lipid rafts (caveolin-1) in its sequence [24]. Here, we showed that, during efferocytosis, PAFR and CD36 co-immunoprecipitated with flotillin-1, which is a protein that is typically found in lipid rafts and is used as a protein marker to detect these microdomains. When LR were disrupted by cholesterol with *β*CD, efferocytosis was reduced, suggesting a requirement of LR integrity for ingestion of AC. Structures like LR permit receptors with a nontransducing short intracytoplasmic portion, like CD36, to come into close proximity with other receptors promoting interactions that allow signalling. These results show that efferocytosis increases the colocalisation of PAFR and CD36 in the macrophage plasma membrane, and that this colocalisation probably happens in the same LR. Although there must be other receptors docked in these LR, these data strongly suggest that PAFR and CD36 interact to achieve optimal efferocytosis.

Regulatory macrophages can be generated by several stimuli such as Fc*γ* and *toll-like* receptor ligands, glucocorticoids, prostaglandins, and apoptotic cells [2, 3, 25]. The major feature of regulatory macrophages is their anti-inflammatory ability due to the production of high levels of IL-10, a potent cytokine with inhibitory effect on immune response, and low levels of IL-12, which has an effect that is opposite to that of IL-10. The balance of IL-10 and IL-12 production has been previously employed to establish the polarisation of macrophage phenotype [5, 26, 27]. We showed here that phagocytosis of apoptotic cells induces more IL-10 than IL-12p40. When macrophages that have ingested apoptotic cells were stimulated with LPS, they produced significantly higher levels of IL-10 than those that had not been exposed to apoptotic cells. This was not observed with IL-12, where the increased levels induced by LPS were not further increased by the phagocytosis of apoptotic cells. Phagocytosis of apoptotic cells by human macrophages even decreased the production of IL-12p70 induced by LPS and IFN-*γ*, as described by Kim et al. [28]. This difference may be explained by the following: they used human macrophages and we used bone marrow-derived murine macrophages; they activated the cells with LPS plus IFN-*γ* and we used only LPS; and they measured the subunit p70 of IL-12, whereas in our case we used p40. In our study, the ratio between IL-10 and IL-12 was increased by efferocytosis, leading to the IL-10^high^/IL-12p40^low^ phenotype, which is characteristic of regulatory macrophages. Moreover, this enhancement of the IL-10/IL-12 ratio was dependent on both PAFR and CD36 engagement, suggesting that both receptors are involved in macrophage polarisation towards a regulatory phenotype. 

In a previous study, we observed that the injection of a subtumorigenic dose of melanoma cells together with apoptotic cells promoted tumour growth and that PAFR antagonists prevented this effect [7]. This suggests that PAFR antagonists, by inhibiting AC recognition, can prevent macrophages from acquiring the regulatory phenotype; and thus the blocking of PAFR during tumour growth could be of therapeutic interest. Another consequence of blocking PAFR is the potential reduction of foam cell formation due to the inhibition of oxLDL uptake, which would be theoretically desirable in atherosclerosis [13, 14]. On the other hand, we showed here that blocking PAFR and/or CD36 reduces the clearance of apoptotic cells, which is an essential mechanism for maintaining homeostasis. The defective clearance of apoptotic cells was associated with some autoimmune and chronic diseases such as systemic lupus erythematosus, type 1 diabetes, chronic obstructive pulmonary disease, and cardiovascular disease [1]. Clearly, the use of agents that block these receptors should be considered with great care. Moreover, some authors propose regulatory macrophages to be used in protocols for immunomodulation to treat inflammatory and autoimmune diseases [29, 30]. In this case, pretreatment of macrophages with ligands of PAFR and/or CD36 might be useful and may increase their drift towards the regulatory phenotype. The present study added to the knowledge of the mechanisms involved in efferocytosis. It would be desirable to further unravel these mechanisms, with the aim of identifying strategies to treat diseases caused by defective efferocytosis and treatments based on immunomodulatory macrophages.

## 5. Conclusion

It can be concluded that the phagocytosis of apoptotic cells engages CD36 and PAFR, possibly in lipid rafts, and this is required for optimal efferocytosis and the establishment of the macrophage regulatory phenotype (IL-10^high^/IL-12p40^low^).
